# Reflux Incidence among Exclusively Breast Milk Fed Infants: Differences of Feeding at Breast versus Pumped Milk

**DOI:** 10.3390/children3040018

**Published:** 2016-10-14

**Authors:** Jennifer Yourkavitch, Sabrina Zadrozny, Valerie L. Flax

**Affiliations:** 1 Department of Epidemiology, University of North Carolina, Chapel Hill, NC 27599, USA; 2 Carolina Population Center, University of North Carolina, Chapel Hill, NC 27516, USA; sabrinaz@unc.edu; 3 Department of Nutrition, University of North Carolina, Chapel Hill, NC 27599, USA; flax@unc.edu

**Keywords:** exclusive breastfeeding, breast milk feeding, reflux

## Abstract

The practice of feeding infants expressed breast milk is increasing in the United States, but the impacts on infant and maternal health are still understudied. This study examines the monthly incidence of regurgitation (gastro-esophageal reflux) in exclusively breast milk fed infants from ages two to six months. Among infants whose mothers participated in the Infant Feeding Practices II Study (IFPS II; 2005–2007), data on reflux and feeding mode were collected by monthly questionnaires. A longitudinal, repeated measures analysis was used, with feeding mode lagged by one month in order to compare reflux incidence among infants fed directly at the breast to infants receiving pumped breast milk. Mothers in both feeding groups had similar characteristics, although a greater proportion feeding at least some pumped milk were primiparous. The number of exclusively breastfed infants decreased steadily between months 2 and 6, although the proportion fed at the breast remained similar over time. An association between feeding mode and reflux incidence was not found; however, the analyses were limited by a small number of reported reflux cases. More studies are needed to further explain the relationship between different feeding modes and infant reflux.

## 1. Introduction

Regurgitation (gastro-esophageal reflux) is a common infant phenomenon [1]. In a prospective evaluation of the natural evolution of regurgitation in healthy infants, regurgitation was highest in the first month, with 73% of infants experiencing it at least once per day, decreasing to 50% at five months of age [2]. Infants regurgitating four or more times per day can often have difficulty with initial weight gain, although most issues are resolved by 12 months of age [2]. However, approximately 7% of infants experience severe reflux, requiring medical care [3]. While the proportion of infants who suffer from reflux is similar among those who are breastfed and those who are formula fed, breastfed infants often have fewer and shorter episodes of reflux [2]. Breastfed infants have more rapid gastric emptying, which can lower median pH values for gastro-esophageal reflux, therefore a lower esophageal pH limits the duration of reflux [4].

The World Health Organization and the American Academy of Pediatrics recommend exclusive breastfeeding (EBF) for the first six months of life [5,6]. An infant who is exclusively breastfed consumes only breast milk, without any additional fluids or foods. EBF confers immediate and long-term benefits to infants and mothers, including reduced risks of diarrheal disease, respiratory illness, and ear infections for infants, and reduced risk of anemia and delayed return to fertility for mothers [5,6,7,8,9,10,11,12,13,14,15,16]. In addition, there is evidence that EBF increases the likelihood of continued breastfeeding beyond six months [7] and there are long-term health benefits of breastfeeding for mothers and infants [17]. Additional benefits for families and society include decreased health care costs [18,19,20], public spending on assistance programs [21], employee absenteeism for parents and related lost income due to sick infants [22]. Breastfeeding also creates a lower environmental burden, by limiting the disposal of bottles, formula packaging and energy demands to create and transport those products [7,11]. Despite the well-documented benefits, the prevalence of EBF for six months still remains low: only 20% within the U.S. [23].

Infants can be exclusively fed breast milk in several ways, including directly at the breast only, pumped breast milk only, or a combination of feedings directly at the breast and pumped breast milk. While breast milk pumping for healthy, term infants is increasing, there is limited evidence about its prevalence and health outcomes [24]. In one study of breast milk expression from a sample of mothers across the U.S., 85% of breastfeeding mothers of infants aged 1.5 to 4.5 months expressed milk at some time and 25% of them had done so on a regular schedule in the previous two weeks [25]. The Affordable Care Act mandates workplace-provided space and time for mothers to pump milk and insurance coverage for breast pumps [26]. In addition, mothers who experience difficulty feeding their infants at the breast or experience delayed lactation onset may also pump their milk [25,27].

There are several factors that potentially differentiate milk that is fed directly at the breast, from milk that has been expressed. The storage and warming of breast milk changes its content, particularly its immunological properties [28,29,30,31,32]. There may be increased bacteria in pumped milk, reduced ascorbic acid concentration, reduced antioxidant activity, hydrolyzed lipids, and lysed immunological cells, although the impact of these changes in milk composition on the health of the infant is not yet fully understood [33]. Bottles can introduce harmful bacteria if the different components of the bottles and of the breast pump are not adequately sanitized [33,34]. One important effect of bottle-feeding is that caregivers may ignore infant cues of satiety. In one study, infants fed breast milk directly at the breast were better able to self-regulate milk intake compared to infants who were fed both by bottle and at the breast, or by bottle only [35]. Additionally, infants fed breast milk in a bottle early in life had a greater likelihood of emptying a bottle later, regardless of whether that bottle contained formula or breast milk [35]. This may be one reason why infants fed from a bottle, whether with breast milk or formula, gained more weight per month than infants fed directly at the breast in another study [36].

An infant’s ability to self-regulate milk consumption has been postulated as a factor associated with reduced reflux, therefore the manner in which infants are fed breast milk may influence the incidence of reflux. Bottle feeding, whether with expressed breast milk or formula, has also been shown to be associated with an increased risk of cough or wheezing in the first year of life, compared to feeding at the breast [37]. Finally, breast milk composition changes during the course of a feeding and as infants age, and some evidence suggests that infants may develop diarrhea and fail to thrive if they receive an imbalanced portion (e.g., if pumped milk is typically lower in fat than milk received at the breast) [33,38].

The association between reflux and method of breast milk feeding has not been investigated. Therefore, the purpose of this study is to examine whether the feeding modality of breast milk—at breast or expressed—is associated with greater incidence of reflux in healthy, term, exclusively-breastfed infants aged two to six months. The question we intend to answer is one of exposure irrelevance, that is, whether feeding mode for exclusively breastfed infants is associated with a greater risk of reflux, which has wide-reaching implications for parent and caregiver counseling and future research.

## 2. Materials and Methods

### 2.1. Population

We conducted a secondary data analysis of the Infant Feeding Practices Study II (IFPS II), a population-based prospective cohort study based in the United States between 2005 and 2007 [39]. The present analysis of IFPS data was exempted from review by the International Review Board of the University of North Carolina, Chapel Hill. The IFPS II study was a joint venture between the U.S. Food and Drug Administration (FDA), the Centers for Disease Control and Prevention (CDC), the Office of Women’s Health (OWH) in the Department of Health and Human Services, the National Institutes of Health (NIH) and the Maternal and Child Health Bureau (MCHB) in the Health Services and Resources Administration (HSRA). The IFPS II cohort comprised of pregnant mothers who were 18 years or older in their third trimester; gave birth to live, singleton infants ≥35 weeks’ gestation; and had infants weighing ≥5 lbs. at birth who did not stay in intensive care longer than three days. Only infants <6 months of age, who reportedly received breast milk exclusively each month from one to six months of age, were eligible for inclusion in this substudy. Analyses were restricted to exclusively breast milk fed infants, to exclude issues related to formula feeding, which is associated with reflux of longer durations [4].

### 2.2. Data Collection

Demographic data were collected in two ways. A demographic questionnaire was routinely sent to consumer opinion panel members by the panel administration, and most mothers’ data came from this panel database. However, if a mother lived in the house of a panel member, but was not the panel member herself, she was sent a separate demographic questionnaire. Women completed a prenatal questionnaire during pregnancy, upon enrollment, and a telephone survey after the birth of their infant. The mother’s age and parity were collected on through the prenatal questionnaire. Follow-up questionnaires were mailed to the women each month, during months 1–7 postpartum, then every 7 weeks until month 12. Monthly questionnaires included data on childcare, sleep, employment, infant feeding patterns and practices, and infant health.

### 2.3. Outcome Assessment

The primary outcome of interest for this study was infant reflux, which was collected by questionnaire at months 2, 3, 4, 5 and 6. Infants were categorized as having reflux at each month if mothers checked a box next to “Reflux” in response to the question asking, “Which of the following problems did your baby have during the past 2 weeks?”

### 2.4. Exposure Assessment

Infant feeding mode was the primary exposure for this study. For each month, infants who were “only fed directly at the breast” were compared to infants who were fed at least “some pumped breast milk”. Infants were categorized as exclusively breastfed at the breast if women were exclusively breastfeeding and replied that infants had not been fed any pumped milk in the past 7 days by providing an answer of zero to the following question: “How many times in the past 7 days was your baby fed pumped breast milk to drink? Include breast milk you expressed in any way as pumped milk.” Infants who were categorized as receiving some pumped milk were exclusively fed breast milk, but had received pumped breast milk one or more times in the past 7 days.

### 2.5. Analysis

**Figure 1:**
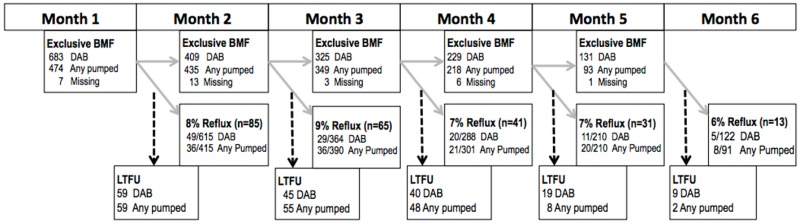
Repeated analysis of the association between exclusive breast milk feeding mode and reflux, with feeding mode lagged one month, for exclusively breast milk fed infants whose mothers participated in the Infant Feeding Practices Survey II (IFPS II) in the U.S., 2005–2007. BMF = breast milk feeding; DAB = direct at breast; LTFU = lost to follow up; Missing observations = crude (n = 127; 3.8%) and adjusted (n = 224; 6.8%).

In order to compare the risk of reflux between infants only fed directly at the breast with infants fed some pumped milk, we used the Generalized Estimating Equations approach (GEE) to log-risk regression with repeated measures for months 2 through 6 (SAS, Version 9.4, Cary, NC, USA). Feeding mode in the past 7 days (the exposure) was lagged one month to ensure that it temporally preceded reflux (outcome) in the past 14 days (Figure 1). The GEE approach conducts a complete case analysis and assumes that all missing data are missing completely at random. All women included in the analysis had no missing outcome data (i.e., reflux in the past 14 days) and were exclusively breastfeeding in the month prior to outcome ascertainment.

We used a directed acyclic graph (DAG) to identify a minimally sufficient set of potential covariates [40]. The following baseline covariates were considered for inclusion: education (college graduate: yes, no), primiparous (yes, no), White, non-Hispanic (yes, no), cesarean delivery (yes, no), enrolled in the Women, Infant and Children (WIC) supplemental program during pregnancy (yes, no), obese body mass index (BMI; >30.0 kg/m^2^: yes, no), infant birthweight in grams (continuous) and if the infant had trouble sucking in the first two weeks (yes, no). The following covariates were originally time-varying, but re-specified to “yes, no”, if experienced at any measured time point, and also considered for inclusion: whether the mother pumped because of sore nipples (yes, no), whether the mother received professional or lay breastfeeding support (yes, no), whether the mother worked in the past four weeks (yes, no), and whether the mother reported smoking at the three-month measure (yes, no). One additional variable, mother’s nativity (country of birth), appeared in our DAG, but did not exist in the dataset, and thus, could not be analyzed.

Those covariates were individually tested in a simple log-risk model with the exposure and the time variable (month). We retained variables meeting these criteria, established a priori: p < 0.20 and <200 additional missing observations. Our final model included these covariates: cesarean delivery, WIC participation, and White/non-Hispanic race/ethnicity.

## 3. Results

Among participants enrolled in IFPS II, this cohort consisted of 1157 infants who were exclusively fed breast milk at the time of the newborn survey and had an outcome measure (reflux incidence) in month 2. Most participants were White/non-Hispanic, college educated and had a normal pre-pregnancy BMI. Mothers’ characteristics were evenly distributed between groups, with the exception of parity (Table 1). A slightly higher proportion of mothers in the WIC supplemental program fed directly from the breast than pumped milk. Neonatal intensive care unit (NICU) stay and infant birth weight were also distributed in similar proportions for both feeding methods.

**Figure 2:**
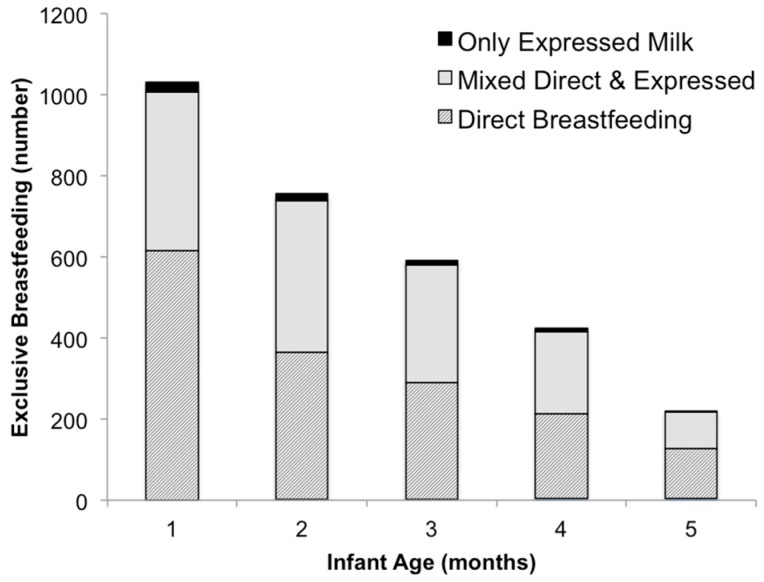
Number of infants who were fed exclusively breast milk each month, and either fed directly at the breast, both directly at the breast and expressed milk, or only expressed breast milk.

The proportion of exclusively breast milk fed infants steadily decreased with each survey cycle, as did the analytic population for each time period. Additionally, the type of feeding mode changed each month, with a greater proportion of infants fed directly at the breast during months 1, 4 and 5 (Figure 2).

Incidence of reflux episodes in this cohort of infants exclusively fed breast milk was low for each month, with 8%, 9%, 7%, 7% and 6% of mothers reporting reflux in their infants at 2, 3, 4, 5 and 6 months, respectively (Figure 1). For months 3, 5, and 6, the proportion of infants experiencing reflux was higher in the group that was fed at least some pumped milk, than in the direct breastfeeding group (Month 3: 9% versus 8%; Month 5: 10% versus 5%; Month 6: 9% versus 4%, respectively), although, overall, no statistically significant effect between feeding modes was found (RR 0.87; 95% C.I. 0.68, 1.12). This estimate did not change, even when adjusting for confounders (RR 0.88; 95% C.I. 0.69, 1.13; Table 2). Of the 3,299 eligible observations (accounting for repeated measures), missing observations were minimal for the crude (n = 127; 3.8%) and adjusted (n = 224; 6.8%) models.

## 4. Discussion

Prior studies of feeding mode and infant health have described some beneficial effects of feeding breast milk directly versus pumped breast milk. For example, direct breastfeeding was correlated with better appetite regulation in childhood, although it did not lead to a different growth pattern [41]. Additionally, incidence of coughing and wheezing was shown to be greater among infants fed bottled breast milk compared to infants fed directly at the breast [37]. Here, we present results from the first study to examine the relationship between breast milk feeding modes and reflux incidence among infants exclusively fed breast milk.

There is some evidence that caregivers’ feeding behavior changes in response to infant reflux [3,42,43]. If bottle feeding promotes caregiver behavior that ignores infant cues of satiety [35], then it is possible that infants fed pumped milk through a bottle may receive a larger volume of breast milk than they would if they had fed directly at the breast, causing reflux. Reflux can cause infant and caregiver distress, leading to changes in infant feeding behavior, including termination of breastfeeding [42]. It is also possible that infants with gastro-esophageal reflux disease (a syndrome characterized by severe gastro-esophageal reflux) have feeding problems [44] which could lead a caregiver to try bottle feeding in an attempt to alleviate an infant’s distress.

For these reasons, we hypothesized that the risk of reflux could increase with expressed milk feedings, possibly due to bacterial contamination, change in milk content, caregiver feeding behavior, or milk removal mechanism (sucking difference and speed of milk flow). The cohort was focused only on infants fed exclusively breastmilk, and while this restriction allowed us to focus on the differential effects of the modes of breast milk feeding, it also reduced our sample size, preventing robust statistical modeling. It should be noted that reflux incidence is common in infancy in the U.S., especially within the first 6 months [2], and since the incidence in our cohort was low, our results may not be generalizable.

Since the objective of this study was to understand whether differences in feeding modes had an effect on reflux, women were included in this study until infants stopped exclusively breastfeeding. Therefore, the effect of breastfeeding cessation, feeding mode, and reflux may be intertwined. In addition, our analysis does not differentiate the reasons participants were LTFU, whether it is because mothers dropped out of the study because they stopped exclusively breast milk feeding, or did not return a survey questionnaire for other reasons. Thus, our study did not differentiate between women who were lost to follow-up and those who stopped exclusively breastfeeding. Additionally, outcome misclassification is a possible limitation since reflux relied on self-reporting, rather than a physician diagnosis. It is also possible that mothers who pumped their breast milk could have had differing reporting styles in reporting reflux incidence, compared to mothers who did not pump. Furthermore, reflux in infants exclusively fed breast milk may be mild or short and may not have been perceived as problematic by their mothers [42] and thus were not reported. Another possible limitation is that the exposure measure, feeding mode, did not differentiate between bottle or cup feeding, so one expressed milk feeding was in the same category as multiple expressed milk feedings. “Lumping” the exposure measure in this way prohibited us from discerning a dose–response relationship, but was necessary in order to have adequate statistical power for the analyses. Exposure was lagged one month to ensure the outcome was observed after exposure to the feeding mode of interest. A one-month lag may be too long between exposure and outcome because reflux occurs within hours after a feeding and caregivers may respond to it by altering feeding practices. We also did not examine interactions with the childcare setting, which could provide information for policies and should be considered in future research. Furthermore, we were not able to examine confounding by some relevant factors due to large amounts of missing data among those potential covariates.

In conclusion, this study did not find a statistically significant association between infant feeding mode and reflux. However, our research provides a useful starting point for future studies. There is much that is unknown regarding the effects of pumped milk feeding on infants’ health. Prospective studies about the differential effects of breast milk feeding modes on maternal and infant health are needed in order to provide appropriate guidance to mothers and caregivers. This issue is becoming especially relevant in the U.S., where more than half of all mothers with a child under one year of age are working [45] and federal policy supports breast milk pumping as a way for working mothers to continue breastfeeding while they are separated from their infants [26].

## Figures and Tables

**Table 1:** Maternal and infant descriptive characteristics for participants who were exclusively breast milk feeding when they completed the newborn questionnaire for the Infant Feeding Practices Survey II (IFPS II), 2005–2007.

	Direct Breastfeeding (n = 683)		Any Pumped Milk Feeding (n = 474)	
	n	(%)	n	(%)
**Maternal Variables**				
**Education**				
High School or Less	91	(14)	49	(11)
Some College	256	(38)	150	(33)
College Graduate or more	318	(48)	252	(56)
**Primiparous**	121	(18)	174	(37)
**Race/Ethnicity**				
White, non-Hispanic	614	(91)	400	(86)
Black, non-Hispanic	6	(1)	13	(3)
Hispanic	25	(4)	29	(6)
Other	27	(4)	23	(5)
**Cesarean Delivery**	151	(22)	127	(27)
**Smoking**	17	(2)	25	(5)
WIC *	170	(25)	90	(19)
Maternal BMI * (pre-pregnancy)				
Underweight (<18.5)	38	(6)	9	(2)
Normal (18.5–24.9)	344	(50)	250	(53)
Overweight (>25.0–29.9)	174	(25)	121	(26)
Obese (>30.0)	122	(18)	89	(19)
				
**Infant Variables**				
NICU * Stay	12	(1)	0	(0)
**Birthweight**				
<2500 g	1	(0)	3	(0)
2500–4000 g	578	(85)	421	(89)
>4000 g	104	(15)	50	(11)

* Abbreviations BMI: body mass index; NICU: neonatal intensive care unit; WIC Women, Infant, and Children supplemental program; Missing values for: education (n = 42); parity (n = 11); race/ethnicity (n = 21); cesarean delivery (n = 1); smoking (n = 1); BMI (n = 10).

**Table 2:** Adjusted risk ratio for feeding mode and reflux among exclusively breast milk fed infants whose mothers participated in IFPS II in the U.S., 2005–2007 *.

Model	Risk Ratio	95% Confidence Interval
Crude	0.87	0. 68–1.12
Adjusted **	0.88	0.69–1.13

* Participants were included in the analysis if they reported that infants were fed only breast milk in each previous month and if there was a response to the question about reflux during each indicated month; ** Adjusted for cesarean delivery, White/non-Hispanic race/ethnicity and participation in the WIC supplemental program.
